# DrugUtilisation: An R Package to Support Drug Utilisation Research Using the OMOP Common Data Model

**DOI:** 10.1002/pds.70433

**Published:** 2026-07-14

**Authors:** Yuchen Guo, Edward Burn, Annika M. Jödicke, Núria Mercadé‐Besora, Kim Lopez‐Güell, Xihang Chen, Mike Du, Xintong Li, Raivo Kolde, Marek Oja, Mees Mosseveld, Katia Verhamme, Julieta Politi, Antonella Delmestri, Wai Yi Man, Peter Rijnbeek, Daniel Prieto‐Alhambra, Martí Català

**Affiliations:** ^1^ Pharmaco‐ and Device Epidemiology Group, Health Data Sciences, Botnar Research Centre, Nuffield Department of Orthopaedics, Rheumatology and Musculoskeletal Sciences (NDORMS) University of Oxford Oxford UK; ^2^ Institute of Computer Science, University of Tartu Tartu Estonia; ^3^ Department of Medical Informatics Erasmus University Medical Center Rotterdam the Netherlands

**Keywords:** drug dose, drug exposure, observational studies, OMOP CDM, treatment adherence, treatment discontinuation, treatment pattern

## Abstract

**Purpose:**

To develop and describe *DrugUtilisation,* an open‐source R package that facilitates drug utilisation studies using data mapped to the OMOP Common Data Model (CDM).

**Methods:**

Core functionalities include creating drug user cohorts, identifying and summarising indications, describing the duration and dose of medication/s and assessing treatment adherence. The package works with packages developed within the DARWIN EU initiative to support study‐specific workflows. We show the package's workflow by analysing the use of simvastatin in three European real‐world databases.

**Results:**

This paper outlines the *DrugUtilisation* package's functions and demonstrates their application with a clinical example of simvastatin use in databases from the United Kingdom, Estonia and the Netherlands. We generated results including cohort counts, indication summaries, measures of dose and duration, as well as publication‐ready tables and figures. We implemented comprehensive unit tests and standardised output format, which ensured consistency across databases and minimised coding errors.

**Conclusion:**

The development of this software allows for researchers to quickly perform common drug utilisation analyses, while also providing the foundation for additional, bespoke study‐specific analyses.

## Key Points

- *DrugUtilisation* is an open‐source R package that standardises drug utilisation analyses on databases mapped to OMOP Common Data Model.
- The package offers comprehensive and user‐friendly functionalities to create cohorts, summarise indications, study dose and duration and analyse treatment adherence and discontinuation.
- The package produces reproducible, standard, publication‐ready study results, tables and figures and supports collaborations by sharing code rather than patient‐level data; therefore improving research reproducibility, methodological rigour, efficiency and consistency.
- Functionalities have been demonstrated and validated on large real‐world datasets from the United Kingdom, Estonia and the Netherlands using simvastatin as a test case.
- Its modular approach integrates very well with other DARWIN EU packages to build custom workflows for different studies.

## Plain Language Summary

Drugs are prescribed in everyday clinical practice and drug utilisation studies help us understand their use. Healthcare databases record prescriptions, refills and related patient information for millions of people, but these data often come in different formats. The OMOP Common Data Model is a shared format that puts different healthcare databases into the same structure so they can be analysed in the same way. We built the *DrugUtilisation* R package, an open‐source R package to conduct drug utilisation analysis of OMOP CDM data. The package has already been used in 62 studies. With only a few lines of code, researchers can define cohorts of drug users, characterise treatment initiation, quantify dose and duration and track changes in use over time. The package also produces publication‐ready tables and figures. As an illustration, we analysed the use of simvastatin (a cholesterol lowering drug) in three national databases from the United Kingdom, Estonia and the Netherlands. *DrugUtilisation* makes the monitoring of medication use faster and easier for researchers, clinicians and regulators.

## 1 Introduction

Drug utilisation is one of the most important areas of observational research in pharmacoepidemiology as it provides insight into the scale and patterns of drug use in the population. Typical analyses include the description and analysis of treatment doses, duration, adherence and switching, with all of them contributing to a better understanding of the drug use pattern [1].

Routinely collected, ‘real‐world’ health data is an important resource for drug utilisation research as it allows us to gain insights on the utilisation of one or more medicines under ‘real‐life’ conditions in routine clinical practice. Importantly, these utilisation patterns can differ substantially from what was observed in the controlled context of pre‐marketing randomised controlled trials, including differences in the treatment duration, doses taken and adherence to the prescribed treatment [2].

Real‐world data (RWD) are increasingly being standardised to common data models, therefore facilitating the application of standard analytical tools against different databases. One of the most commonly used data models is the Observational Medical Outcomes Partnership (OMOP) Common Data Model (CDM), a person‐centric data model which standardises both the structure and the vocabulary of the mapped datasets [3]. The OMOP CDM has been used for a wide range of research projects and applications; however, its use for standardised drug utilisation research has been somewhat limited [4, 5]. Recently, substantial efforts have gone into improving the methodology for calculating daily drug doses in RWD mapped to the OMOP CDM from various international databases [6]. Subsequently, the development of standardised tools to support drug utilisation analyses was needed.

We therefore developed the ‘DrugUtilisation’ R package, which aims to facilitate drug utilisation analyses using data mapped to the OMOP CDM. The package provides a range of well‐tested functionalities for drug utilisation analyses. It was developed with input from the European Medicines Agency within the DARWIN EU initiative and works with other DARWIN EU packages to support study‐specific workflows. We demonstrate the package's functionality through an example drug utilisation study on simvastatin use using RWD from the United Kingdom, Estonia and the Netherlands.

## 2 Methods

### 2.1 The DrugUtilisation R Package

The DrugUtilisation R package was developed to support drug utilisation studies commissioned by the European Medicines Agency (EMA) within the context of the DARWIN EU initiative [7]. DARWIN EU builds on a network of RWD sources mapped to the OMOP CDM and a prespecified ‘catalogue of standard analytics’ including descriptive analyses for drug utilisation [7]. The package was co‐developed with epidemiologists and data scientists to support these analyses while offering intuitive user interfaces. It complements other R packages designed for OMOP‐mapped data, including those for estimating incidence and prevalence [8], characterising patient populations [9] and survival analyses [10].

DrugUtilisation was developed according to R package best practices [11] and created with the support of *devtools* and *usethis* [12, 13] and with unit testing via *testthat* [14]. Its core dependencies include *dplyr* and *dbplyr* for SQL translation and packages tailored for OMOP CDM: *omopgenerics* [15] for defining common classes and methods, *CDMConnector* [16] for connecting to CDM tables and *PatientProfiles* [17] for working with patient‐level data. All exported functions are covered by comprehensive unit tests, which validate functionality across various inputs and database platforms (PostgreSQL, Microsoft SQL Server, Amazon Redshift, Snowflake, Spark SQL, DuckDB …). These tests are executed both locally and through continuous integration with GitHub Actions.

### 2.2 Main Functionalities

#### 2.2.1 Drug User Cohort Creation

**FIGURE 1:**
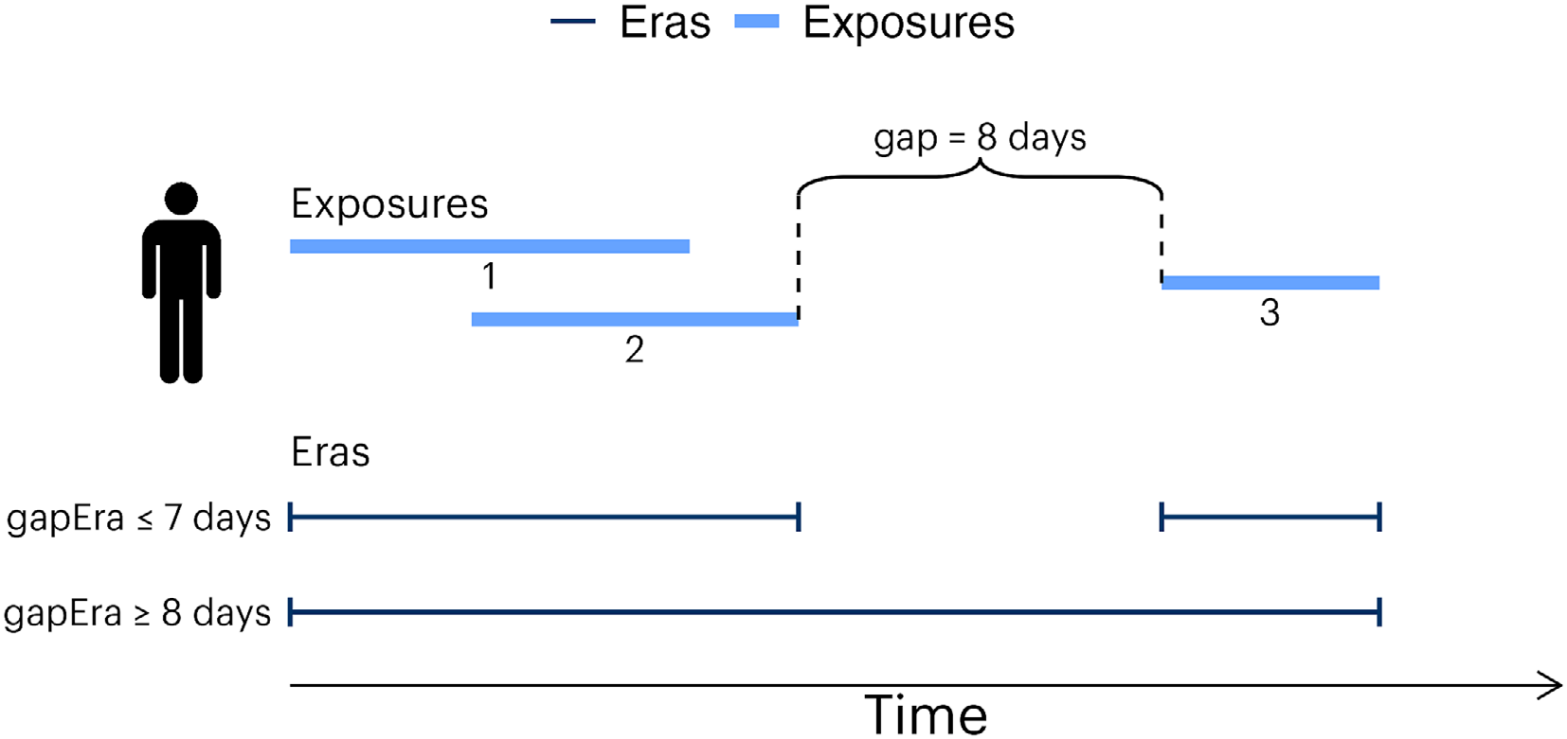
For this illustration, ‘Exposure’ is defined as the estimated duration of a prescription/dispensation. Exposures collapse at cohort creation stage. Overlapping drug records are always collapsed in a common era (exposures 1 and 2). Exposure 3 is merged with 1 and 2 into a single continuous era depending on gapEra parameter; if gapEra ≤ 7 days two separate eras are created as the distance between the end of exposure 2 and start of exposure 3 is 8 days; otherwise, if gapEra ≥ 8 days, a single era episode is created.

The creation of drug user cohort is the first step of any drug utilisation study. Our package supports the generation of drug user cohorts (as defined by omopgenerics [15]) based on different input types and configurable parameters. Three functions allow the user to create drug cohorts based on concept sets, ingredients or using the Anatomical Therapeutic Chemical (ATC) classification system (a WHO‐maintained hierarchical drug classification system), respectively: *generateDrugUtilisationCohortSet*, *generateIngredientCohortSet* and *generateAtcCohortSet*. A key parameter to specify when creating drug cohorts is the *gapEra*, which specifies the maximum number of days allowed between repeated drug prescriptions to be considered continuous drug exposures, called ‘treatment era’. Drug records separated by a number of days ≤ *gapEra* are collapsed into a single cohort record (Figure 1).

Additional restrictions can be applied to these cohorts, including requiring a washout period prior to cohort entry (*requirePriorDrugWashout*), restricting to the first drug record ever (*requireIsFirstDrugEntry*), requiring a minimum of prior observation in the database (*requireObservationBeforeDrug*) and restricting to drug records within a specified period (*requireDrugInDateRange*). These inclusion criteria are not commutable operations (i.e., operations whose outcome depends on the order in which they are applied) and the order of their execution must be chosen carefully by the user [18]. We report cohort attrition, the number and proportion removed at each step, to support traceability, reproducibility and validation and to populate the study flowchart.

#### 2.2.2 Indication

Associating prescriptions with their corresponding indications is important to review the appropriateness of drug use, understand off‐label use and support effective patient care, as well as healthcare policy planning and decision‐making [19]. However, in most RWD databases, drug records do not have an associated indication recorded. Therefore, potential indications are often obtained via proxies based on recorded diagnoses immediately before/after therapy initiation. DrugUtilisation enables users to summarise potential indications from cohorts of conditions of interest using the *summariseIndication* function. These condition cohorts must be pre‐defined and created separately using other available packages [20].

**FIGURE 2:**
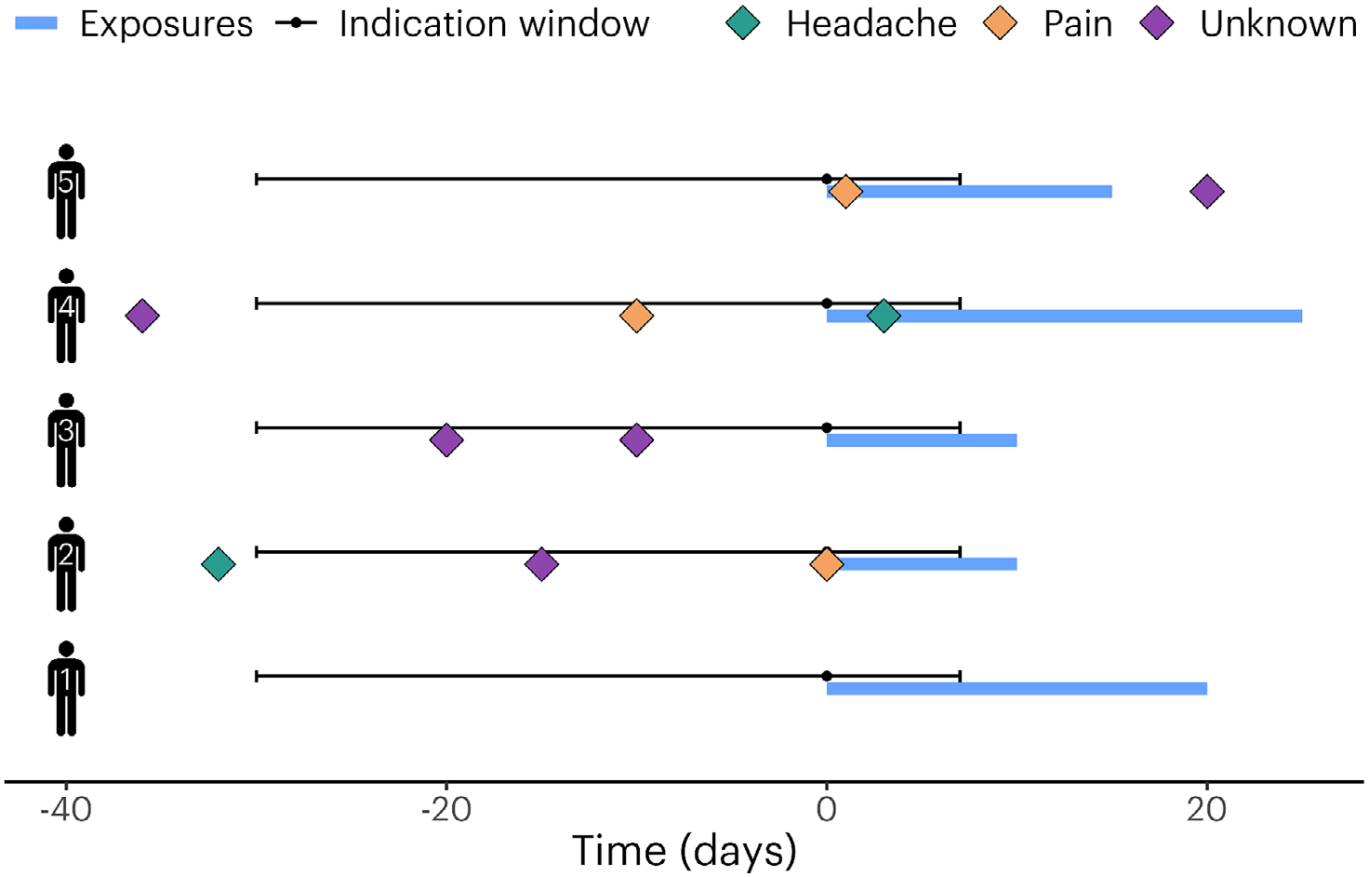
Potential indications are assessed based on records of conditions of interest in pre‐specified time windows prior to the index data (drug start); any record falling inside the indication window will be considered an indication. In this figure, the indication window is [−30, 7].

Potential indications are identified based on the recording (presence/absence) of the pre‐defined conditions of interest in a time window with respect to the index date (drug start). Indication time window is usually set to only index date ([0, 0]) but can be expanded to include other windows of interest for the user, such as to the week or the month before index date ([−30, 0], [−7, 0], respectively). The percentage of each potential indication is calculated per time window. Percentages can be reported in mutually exclusive categories or overlapping categories (see *mutuallyExclusive* parameter). When *mutuallyExclusive = FALSE*, individuals can contribute to more than one indication category if multiple conditions are recorded within the specified time window. When *mutuallyExclusive = TRUE*, each individual is assigned to a single unique category, with additional combination categories created for individuals who have multiple recorded conditions. For instance, in the example illustrated in Figure 2, we would report the indications as follows: 20% headache, 60% pain and 40% no indication for *mutuallyExclusive = FALSE* and 0% headache, 20% headache and pain, 40% pain and 40% no indication for *mutuallyExclusive = TRUE*.

In addition, an ‘unknown’ indication can be reported: people with an unknown indication do not have a record of any of the pre‐specified conditions of interest, but they do have a record of any other condition/observation recorded in, for example, ‘condition_occurrence’ or ‘observation’ tables in the respective time window of interest (‘*unknownIndicationTable’*). In the example of Figure 2, the percentage of unknown indication would then be 20%, leaving only 20% of individuals with no indication.

#### 2.2.3 Dosage and Drug Utilisation

Our package allows to describe user's drug utilisation by summarising several variables related to treatment duration using the *summariseDrugUtilisation* function: number prescriptions/dispensations (referred to as ‘exposures’ in the package), number of treatment eras, days prescribed (total number of days covered by a recorded prescription), days exposed (total number of days within a treatment era incl. treatment gaps < *gapEra*). Additional variables include time to first record, duration of initial exposure (first record), initial quantity dispensed and cumulative quantity. Definitions for all variables are included in Appendix A1.

The exposures included in the analysis are determined by the *conceptSet* parameter, where a concept set of RXNorm (the standard drug vocabulary in OMOP, providing normalised names and identifiers for medications) codes for the treatment of interest is specified. An ingredient must always be provided in the *ingredientConceptId* parameter to get information related to dosage as dose is always calculated with respect to an ingredient. This avoids ambiguity in cases where a drug contains multiple ingredients and ensures the correct strength information and dose formulas are applied. Daily dose was calculated using a standardised algorithm based on quantity and duration (end date−start date + 1) from the drug exposure table, combined with concentration information from the drug strength table. The method applies four formula patterns tailored to different drug formulations: fixed amount, concentration‐based, time‐based without denominator and time‐based with denominator. This approach has been validated by showing that the resulting median daily doses are generally consistent with World Health Organization Defined Daily Doses, while achieving high coverage across drug records (> 85% in most datasets) [6].

The *restrictIncident* option controls whether to include only incident prescriptions (default *restrictIncident* = TRUE) or can be set to FALSE to include prevalent treatments that started before the index date of the cohort. If the cohort was created as described in Section 2.2.1, then all treatments will be incident and the parameter would be redundant; when a cohort is created based on conditions or another drug, then *restrictIncident* will have an impact.

**FIGURE 3:**
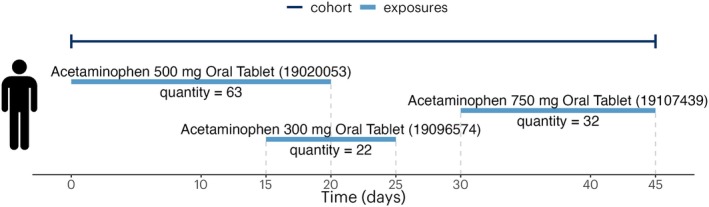
Dosage and drug use calculation.

Figure 3 illustrates the calculation of number of days exposed [with *gapEra* = 0] (42 days), the number of days prescribed (48 days) and the cumulative dose (62 100 mg) for three acetaminophen prescriptions/dispensations. Calculations for all other variables are provided in Appendix A1.

#### 2.2.4 Treatment Adherence

**FIGURE 4:**
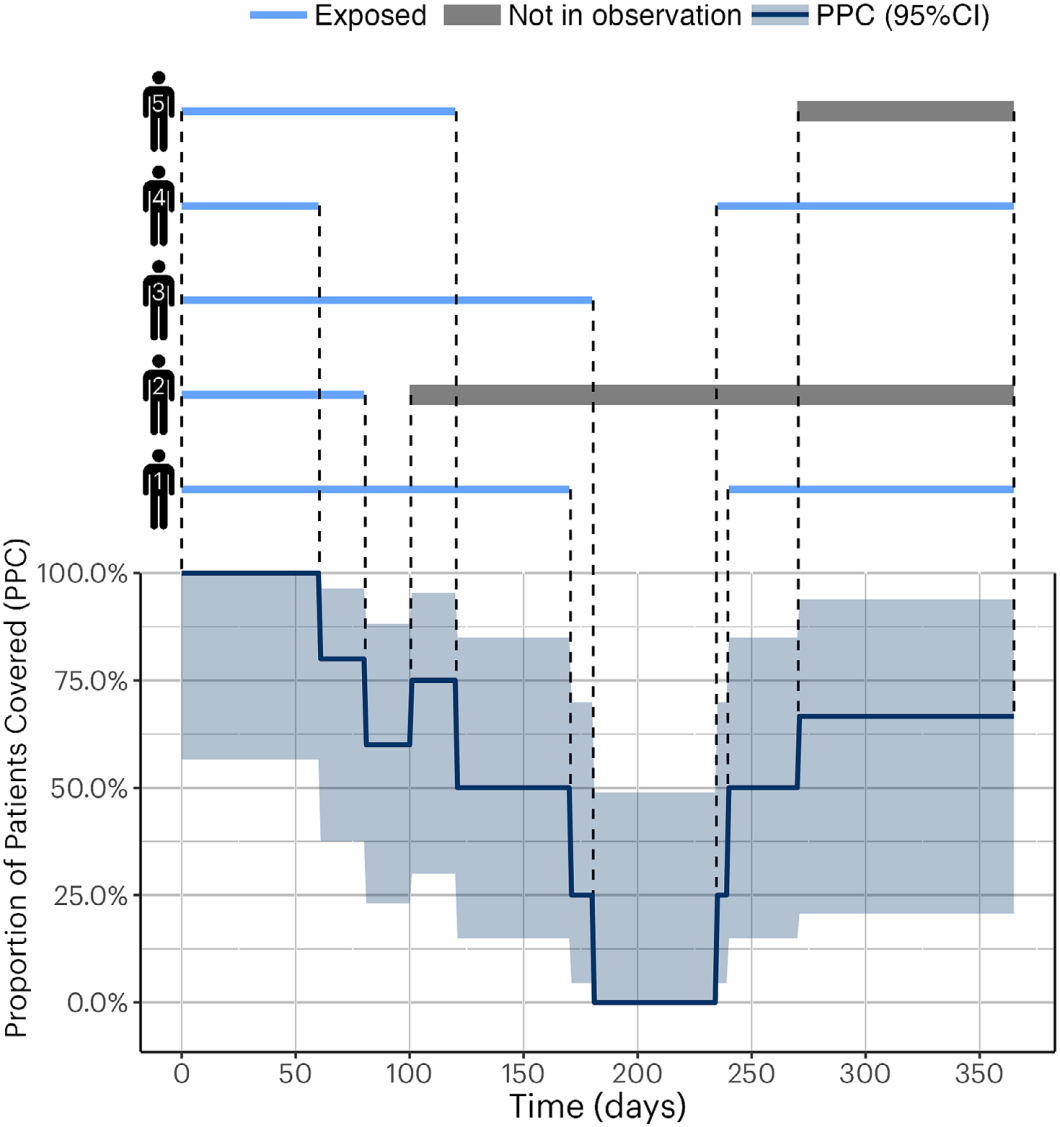
Proportion of patients covered (PPC). PPC of five individuals over time; the numerator is the number of patients who have medication on hand (‘covered’); the denominator is the number of patients under observation that day. PPC is calculated daily and individuals that stop the treatment can contribute to the numerator in subsequent (future) days. Individuals that are not in observation do not contribute to the denominator.

The *summariseProportionOfPatientsCovered()* function quantifies adherence by calculating the proportion of patients [21] under treatment over a follow‐up period defined by *followUpDays*. The percentage of exposed individuals on each day during follow‐up is reported as shown in (Figure 4), thus providing insight into treatment adherence over time. Individuals contribute to the numerator while exposed and in the denominator while they are in observation.

#### 2.2.5 Treatment Switching and Restart

In drug utilisation studies, analysing drug restart or switching behaviours after treatment discontinuation is important. In our package, the *summariseDrugRestart()* function analyses what happens once a treatment episode ends within a number of days since discontinuation (*followUpDays*). This function categorises events into four unique groups:

- Restarting the original treatment only.
- Switching to an alternative treatment only.
- Both restarting the original treatment and switching to another.
- Fully discontinuing treatment (neither restarting the original treatment nor starting any alternative treatment).

**FIGURE 5:**
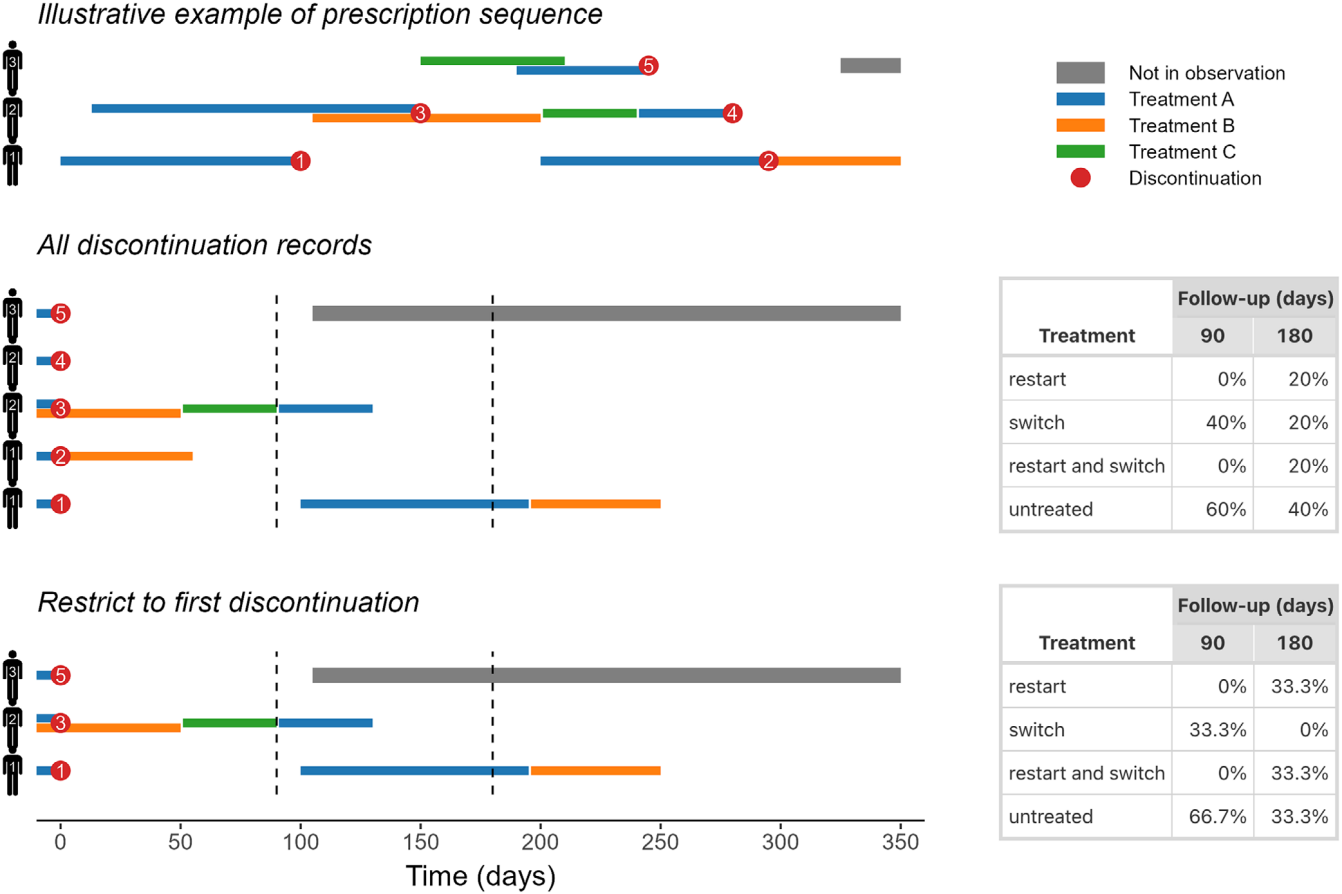
Treatment switching and restart after discontinuation. This example analyses discontinuation of Treatment A. Five discontinuations are identified, shown as red points. Treatments B and C are considered alternative treatments for switching, while subsequent Treatment A represents restart. The top panel shows the prescription sequences for patients 1, 2 and 3 over time. The middle panel shows the discontinuation analysis using all discontinuation records (restrictToFirstDiscontinuation = FALSE). The bottom panel shows the analysis restricted to the first discontinuation per patient (restrictToFirstDiscontinuation = TRUE). The tables show the proportion of patients classified as restart, switch, restart and switch or untreated within each follow‐up window.

The package user can opt to include all exposures and discontinuations or restrict to only the first discontinuation (*restrictToFirstDiscontinuation* parameter). The example shown in Figure 5 illustrates discontinuation of a treatment A.

#### 2.2.6 Summarise Treatments Across Time Windows

**FIGURE 6:**
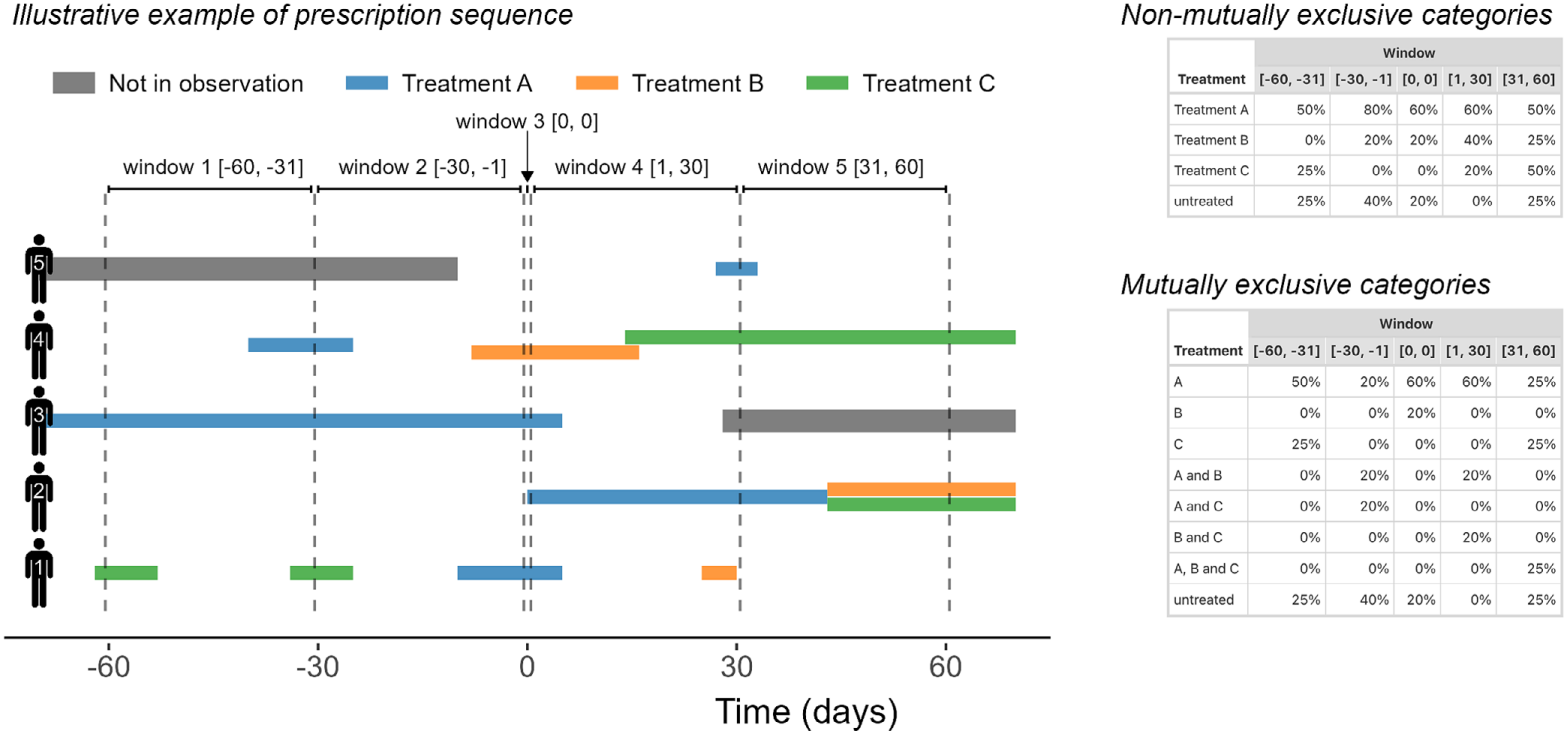
Summarising treatments over time. Time is shown in days relative to the index date. The left panel shows treatments (A, B and C) received by individuals 1, 2, 3, 4 and 5 over time. The upper‐right table shows the percentage of individuals receiving each treatment in each time window using non‐mutually exclusive categories (mutuallyExclusive = FALSE). The lower‐right table shows the percentage of individuals assigned to each mutually exclusive treatment category in each time window (mutuallyExclusive = TRUE).

Summarising drug prescriptions/dispensation in the immediate time before/after a new treatment initiation can be useful for identifying comedications, treatment patterns over time or treatments initiated after discontinuation. This can be done using time windows with *summariseTreatment()*, where the user specifies an index date, one or more time windows of interest and treatment cohorts. The percentage of individuals in observation taking each of the pre‐specified treatments in each time window is reported. Treatments can be reported in mutually exclusive categories, similar to those illustrated for the *summariseIndication()* function (Figure 6).

#### 2.2.7 Other Functionalities

In addition to the functionalities above, the DrugUtilisation package contains functionalities for visualisation. Each summarise* function contains its analogous table* function to display results in a table and its plot* function to display results in a figure, for example, *summariseIndication*(), *tableIndication()* and *plotIndication()*. The package also allows users to add variables—such as indications, exposure, duration and dose related metrics—to the patient table through functions that start with add* (e.g., *addDrugUtilisation()*, *addIndication()*, …), which then facilitates the stratification of drug utilisation analyses for these variables. Finally, the package provides additional utility functions, such as *summariseDoseCoverage()* which estimates the proportion of overall exposure attributable to a specified active ingredient.

### 2.3 Use Case: Using the DrugUtilisation R Package to Characterise ‘Simvastatin’ Use in the UK, Estonia and Netherlands

To ensure that the DrugUtilisation package meets the requisite performance for use on large health care datasets and to ensure the face validity of results across multiple international databases, we conducted a use case study. We analysed the use of simvastatin, a HMG‐CoA reductase inhibitor, for the treatment of hypercholesterolemia and the prevention of cardiovascular events. We based our study on three RWD sources mapped to OMOP, namely MAITT [national healthcare claims and discharge summaries from Estonia, covering 150 000 patients (a 10% national sample)] [22], Clinical Practice Research Datalink (CPRD GOLD) [primary care electronic health records (EHR) from the United Kingdom, covering 3 million currently registered patients] [23] and the Integrated Primary Care Information database (IPCI) [primary care EHR from The Netherlands, covering 2.5 million patients] [24]. Both CPRD GOLD and IPCI are broadly representative of the UK and Dutch population in terms of age and sex, respectively.

We built a new‐user simvastatin cohort by applying: (i) a gap of 30 days was applied to merge prescription records into a single treatment era. (ii) a 365‐day washout with no simvastatin exposure before the index date, (iii) ≥ 365 days of prior observation, (iv) restriction of index dates to the prespecified study period and (iv) getting only first exposure per person. We summarised drug utilisation characteristics including number of prescriptions/dispensations, treatment duration and dose and characterised potential treatment indications and estimated adherence, measured by the proportion of patients covered over a 12‐month follow‐up. Finally, we assessed treatment changes following discontinuation by describing patients who restarted simvastatin, switched to an alternative treatment (different statin, ezetimibe, alirocumab or evolocumab) or remained untreated. This use case illustrates the package's flexibility for conducting comprehensive and reproducible drug utilisation studies using OMOP CDM data.

The full study code is available here: https://github.com/darwin‐eu‐studies/DrugUtilisationArticle.

## 3 Results

### 3.1 DrugUtilisation

The first version of the DrugUtilisation R package, which requires *R* ≥ 4.1, was released on CRAN on 19th May 2023 [25]. As of 28th October 2025, the package is available with Version 1.0.4 and has been downloaded 17 681 times and has been used in 62 DARWIN‐EU studies. The code is publicly available in GitHub (https://github.com/darwin‐eu/DrugUtilisation) with a website providing the package documentation, examples and vignettes (https://darwin‐eu.github.io/DrugUtilisation/). The package has a 98% and 77% code coverage in tests and vignettes, respectively, ensuring that tests and examples cover a wide range of use cases.

### 3.2 Use Case: Simvastatin Utilisation

The study code was run locally in the three contributing databases. Complete drug utilisation results were obtained within 2.22 h for MAITT, 1.22 h for IPCI and 29.85 h for CPRD GOLD, reflecting differences in database scale and site‐specific computing environment. All results are available in an interactive web application (https://data.darwin‐eu.org/DrugUtilisationArticle/). Results from drug utilisation analyses on the number of prescriptions, indication, treatment duration, adherence and switching are presented in the following section.

#### 3.2.1 Number of Simvastatin Users and Prescriptions

A total of 1 634 717 simvastatin users were identified (1 404 985 in CPRD GOLD, 3744 in MAITT and 225 988 in IPCI). Table 1 summarises the number of new users of simvastatin (after application of inclusion criteria) showing the median number of exposures, days exposed and cumulative dose, stratified by age group and data source. Simvastatin was predominantly prescribed among people aged 60 and older. In CPRD GOLD, the median number of prescriptions increased with age, with up to 6 (2–21) prescriptions among people aged ≥ 80 years, indicating more repeat prescriptions in older patients.

**TABLE 1:** Number of subjects, exposures, days exposed and cumulative dose across different databases for new users of simvastatin.

Variable name	Estimate name	Database		
		IPCI	MAITT	CPRD GOLD
0 to 19				
Number subjects	*N*	88	< 5	173
Number exposures	Median (Q25–Q75)	2 (1–4)	—	3 (1–8)
Days exposed	Median (Q25–Q75)	112 (60–296)	—	105 (30–336)
Cumulative dose milligram	Median (Q25–Q75)	2400 (900–4200)	—	2240 (1120–6360)
20 to 39				
Number subjects	*N*	2061	32	9898
Number exposures	Median (Q25–Q75)	2 (1–4)	1 (1–3)	3 (1–8)
Days exposed	Median (Q25–Q75)	109 (60–286)	60 (30–139)	102 (56–299)
Cumulative dose milligram	Median (Q25–Q75)	3600 (1800–10 800)	1200 (600–2230)	3360 (1338‐ 10 080)
40 to 59				
Number subjects	*N*	28 694	462	126 428
Number exposures	Median (Q25–Q75)	2 (1–6)	1 (1–3)	4 (1–13)
Days exposed	Median (Q25–Q75)	148 (90–427)	60 (60–127)	145 (56–506)
Cumulative dose milligram	Median (Q25–Q75)	4800 (1800–15 000)	1200 (600–2400)	4760 (2240–17 360)
60 to 79				
Number subjects	*N*	47 292	1044	194 881
Number exposures	Median (Q25–Q75)	3 (1–7)	1 (1–3)	5 (2–18)
Days exposed	Median (Q25–Q75)	166 (90–479)	60 (60–145)	197 (56–693)
Cumulative dose milligram	Median (Q25–Q75)	5400 (1800–16 800)	1200 (832.50–3000)	6720 (2240–22 400)
80 to 150				
Number subjects	*N*	8299	244	38 126
Number exposures	Median (Q25–Q75)	2 (1–6)	1 (1–3)	6 (2–21)
Days exposed	Median (Q25–Q75)	102 (46–365)	60 (60–144)	208 (56–650)
Cumulative dose milligram	Median (Q25–Q75)	3600 (1200–12 600)	1200 (600–3600)	6720 (2240–21 280)
Overall				
Number subjects	*N*	86 434	1783	369 506
Number exposures	Median (Q25–Q75)	3 (1–6)	1 (1–3)	5 (1–16)
Days exposed	Median (Q25–Q75)	150 (90–447)	60 (60–140)	174 (56–611)
Cumulative dose milligram	Median (Q25–Q75)	4840 (1800–15 600)	1200 (600–2920)	6160 (2240–20 160)

#### 3.2.2 Indication

**FIGURE 7:**
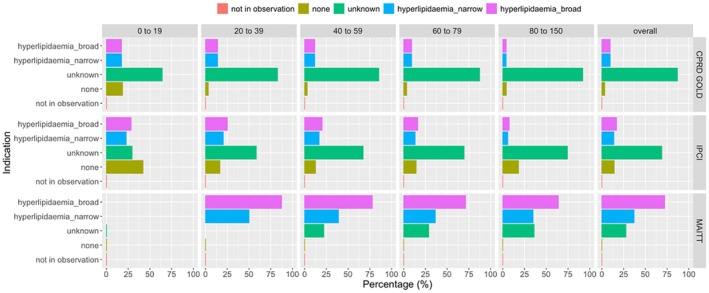
Recording of conditions of interest related to potential indication in the 30 days before the index date, shown by data source (rows) and age group (columns). Categories are: hyperlipidaemia_narrow (specific hyperlipidaemia codes), hyperlipidaemia_broad (broader lipid‐disorder codes), unknown (condition/observation recorded but not included in pre‐specified indication cohorts), none (no condition/observation recorded) and not in observation (individual was not under continuous follow‐up for at least 1 day of the look‐back period). Codelists for the broad and narrow definitions are available in the study code repository on GitHub.

We defined ‘hyperlipidaemia’ as the indication of interest recorded within 30 days before the simvastatin prescription, with two different definitions (‘narrow’ definition: specific hyperlipidaemia diagnosis codes; ‘broad’ definition: diagnosis codes related to lipid‐disorders). Figure 7 illustrates that in both CPRD GOLD and IPCI, the proportion of people with a record of hyperlipidaemia was low (ranging between 17%–28% in the age group ≤ 18 and around 9%–16% in people aged 60–79). In contrast, a high proportion of new simvastatin users in MAITT had a coded hyperlipidaemia diagnosis in the month before treatment start (ranging between 64%–88% for the ‘broad’ and 35%–50% for the narrow definition).

#### 3.2.3 Treatment Duration

The duration of the first continuous simvastatin treatment episode (‘days exposed’) for the new users of simvastatin, stratified by age group, is shown in Table 1. The median and interquartile ranges of exposure duration increase with age. Treatment duration was generally shorter in MAITT compared to the primary care databases CPRD GOLD and IPCI.

#### 3.2.4 Treatment Adherence

**FIGURE 8:**
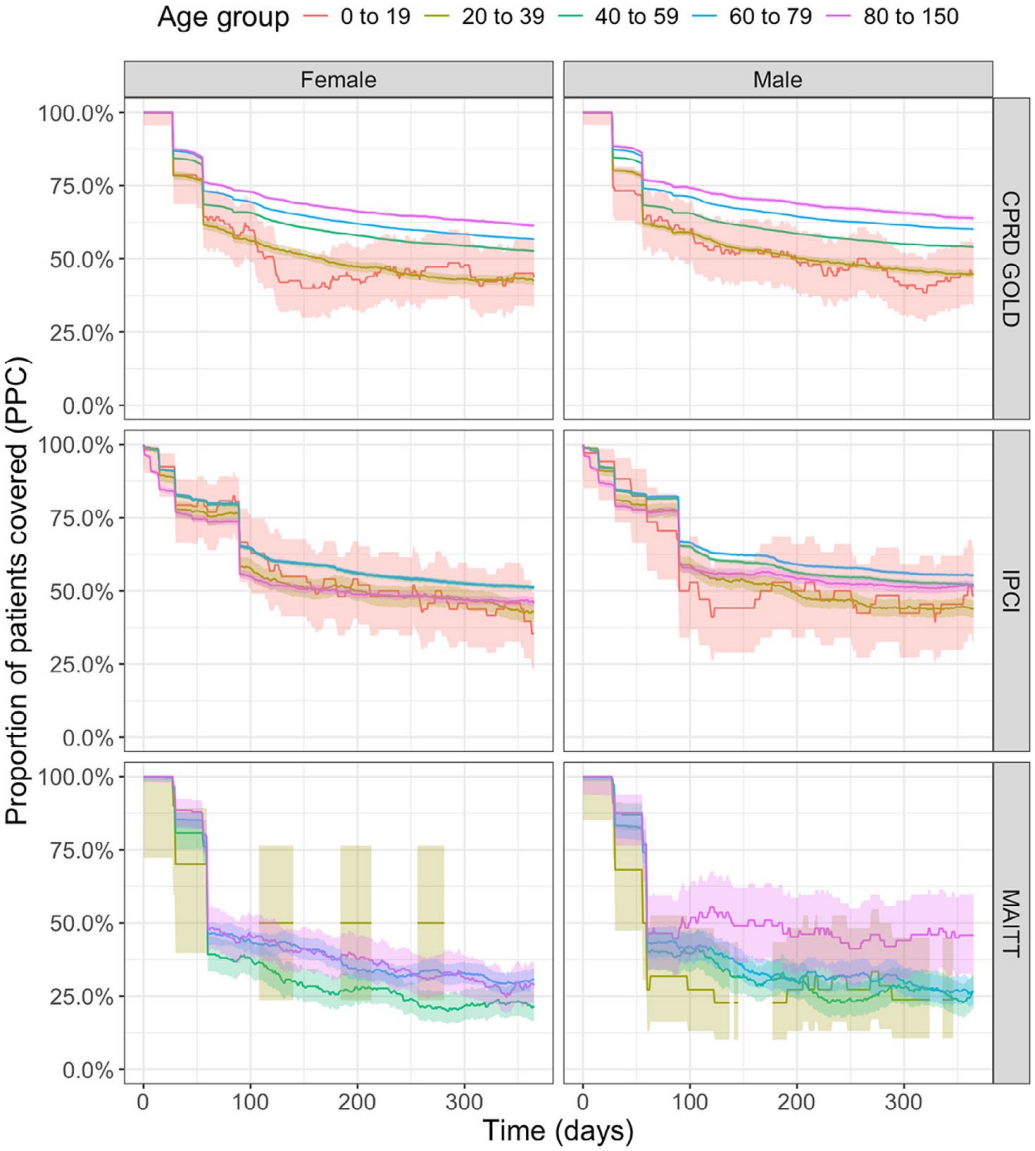
Adherence to simvastatin treatment displayed as proportion of patients covered, stratified by age groups, sex and database.

Figure 8 presents the proportion of patients covered (PPC) over time, illustrating adherence to simvastatin therapy by age group and sex for the new users of simvastatin. In all databases, younger age groups demonstrate a more rapid decline in coverage, whereas coverage in older groups tends to persist longer. CPRD GOLD and IPCI show more gradual declines compared to MAITT, where early treatment discontinuation is more common. Similar adherence patterns are observed in male and female groups.

#### 3.2.5 Treatment Switching and Restart

Treatment episodes are first merged into eras using the *gapEra*; restarts and switches are counted only when a gap exceeds *gapEra*, with shorter gaps treated as continuous use. In all databases, the proportion of new users of simvastatin who resumed simvastatin increased over time: by 1 year, 47% of MAITT users and approximately 42% of CPRD GOLD and IPCI users had restarted therapy. Switching to an alternative agent was most common in CPRD GOLD, reaching 18% at 1 year and least frequent in MAITT (10%). Combined ‘restart and switch’ events remained uncommon throughout (≤ 5% everywhere). Conversely, the share of individuals who received no further therapy (‘untreated’) declined with longer follow‐up but still ranged from 35% in IPCI to 40% in MAITT at 360 days. Men and women show similar patterns in restarting or switching treatment, with men generally having slightly higher restart rates across datasets and time point as seen in Table 2. Results for the rest of stratifications are available in the web application.

**TABLE 2:** Result for patients' treatment restart, switch, restart and switch, or untreated for each database‐sex combination within each time window (90, 180, 270, 360 days).

Treatment	CDM name					
	IPCI		MAITT		CPRD GOLD	
	Sex					
	Female	Male	Female	Male	Female	Male
Drug restart in 90 days						
Restart	27.79%	29.42%	30.23%	29.81%	34.46%	37.13%
Switch	16.19%	14.78%	6.33%	7.14%	17.09%	15.75%
Restart and switch	1.37%	1.60%	0.88%	0.93%	0.93%	0.86%
Untreated	54.64%	54.20%	62.57%	62.11%	47.52%	46.26%
Drug restart in 180 days						
Restart	37.02%	39.45%	40.69%	41.15%	39.02%	42.47%
Switch	16.99%	15.32%	7.47%	9.01%	17.92%	16.42%
Restart and switch	2.76%	3.02%	1.58%	1.86%	2.19%	2.05%
Untreated	43.24%	42.21%	50.26%	47.98%	40.88%	39.06%
Drug restart in 270 days						
Restart	39.79%	42.47%	44.55%	44.41%	39.80%	43.56%
Switch	17.52%	15.63%	8.35%	9.32%	18.41%	16.88%
Restart and switch	3.84%	4.20%	2.64%	3.11%	3.33%	3.16%
Untreated	38.85%	37.70%	44.46%	43.17%	38.46%	36.41%
Drug restart in 360 days						
Restart	41.47%	44.40%	47.01%	47.20%	39.89%	43.82%
Switch	17.91%	15.92%	9.49%	9.94%	18.81%	17.22%
Restart and switch	4.92%	5.21%	2.99%	4.35%	4.34%	4.19%
Untreated	35.70%	34.48%	40.51%	38.51%	36.96%	34.78%

## 4 Discussion

The DrugUtilisation R package was developed to facilitate drug utilisation analyses using data mapped to the OMOP CDM. Its design and output were based on the pre‐specified DARWIN EU(r) catalogue of standard analytics, with input from the European Medicines Agency. DrugUtilisation provides functionality to create drug user cohorts, describe indications and drug use, summarise adherence, analyse treatment restart and switching and describe comedication use in time windows around treatment initiation date.

The package works alongside other complementary packages that provide functionality for conducting RWD analyses, in the context of DARWIN EU and beyond. For example, once a cohort of drug users is created, we could conduct incidence analysis using the IncidencePrevalence package [8], characterise the drug user using the CohortCharacteristics package [9] or analyse discontinuation as a survival analysis using CohortSurvival [10].

The DrugUtilisation package is built for OMOP CDM by design. Basing analyses on a common data model simplifies interfaces, standardises outputs and allows code to be reused across datasets and sites. Without a CDM, drug‐utilisation studies typically require bespoke extractions and a data model for each research question, limiting comparability and reproducibility. Where datasets are not yet mapped, we prefer mapping data to OMOP CDM over repeated study‐specific preparation, as this enables consistent implementation and auditable, reusable analyses. This approach does, however, inherit certain constraints from the OMOP framework. Most notably, drug records are defined using concept sets drawn from standardised vocabularies, which means that treatments not adequately represented in those vocabularies (or mapped inconsistently across sites) may be missed or incompletely captured. Researchers should therefore verify the coverage and granularity of relevant concept sets using some of the available diagnostics tools [26, 27]. An inherent limitation of the package is that users are expected to have a working knowledge of the R programming language to use it. This prerequisite may pose a barrier for some researchers, particularly those from non‐technical backgrounds. In both current and future developments, we will focus on enhancing user‐friendliness and providing comprehensive documentation. This includes the creation of a detailed user manual and the production of YouTube video workshops to broaden the package's accessibility within the research community [28].

Another consideration is that the package reports drug utilisation measures based on the information recorded in the source data and does not attempt to infer pharmacologically equivalent exposure across formulations or routes of administration. Therefore, dose‐based analyses should be interpreted carefully, as cumulative dose estimates may be sensitive to changes in route of administration due to differences in absorption and bioavailability across formulations.

Furthermore, while the DrugUtilisation package supports different functionalities for drug utilisation research, users should be aware of potential limitations associated with the quality and completeness of the RWD they have access to. Variability in data quality across different databases mapped to the OMOP CDM may influence the results of analyses conducted with the package. Consequently, a thorough assessment of data quality is essential to ensure robust research outcomes using dedicated packages like PhenotypeR or DrugExposureDiagnostics [26, 27, 29]. This is particularly relevant for drug exposure duration, as the package assumes that drug exposure end dates are known or can be derived from the available data. While this assumption is common in drug utilisation research, it should be noted that drug exposure end date is not a mandatory field in the OMOP CDM and, when missing, the package assumes that it is equal to the drug exposure start date. Estimating treatment duration may therefore require additional assumptions or methodological approaches based on information such as days supply, quantity dispensed, prescribed dose or gaps between prescriptions. Several methods and tools have been proposed to support exposure duration estimation in Drug Utilisation studies [30].

## 5 Conclusion

The DrugUtilisation R package provides a user‐friendly and well‐tested tool to support the conduct of drug utilisation studies using data mapped to the OMOP CDM. It is part of a growing ecosystem of R packages and tools that facilitate the development of epidemiological studies using OMOP mapped data.

## Author Contributions

M.C., Y.G., E.B., K.L.‐G., N.M.‐B., X.C., M.D. and X.L. developed the DrugUtilisation package. Y.G. and M.C. created the analysis script. M.C., Y.G., R.K., M.O., M.M., K.V. applied the script to their own datasets and were responsible for executing the study. A.D. and W.Y.M. mapped CPRD GOLD data to OMOP CDM. E.B. drafted the first version of the manuscript. Y.G., M.C. and A.M.J. revised it and produced the final version. A.M.J., P.R., D.P.‐A. and M.C. contributed to project management and result interpretation. All authors reviewed results, revised the manuscript critically, approved the final version for submission, and agreed to be accountable for the integrity of the work.

## Funding

The European Medicines Agency funded the development of the *DrugUtilisation* package within the framework of the Data Analysis and Real‐World Interrogation Network (DARWIN EU). The content of this manuscript reflects solely the views of the DARWIN EU Coordination Centre and should not be understood as representing the opinions of the European Medicines Agency or the European Medicines Regulatory Network.

## Ethics Statement

The protocol for this research was approved by CPRD's Research Data Governance Process (protocol number 24_004294), the IPCI review board (protocol number 11/2022) and for MAITT data: Research Ethics Committee of the University of Tartu (approval 300/T‐23) and the Estonian Committee on Bioethics and Human Research (approval 1.1–12/653).

## Conflicts of Interest

D.P.‐A.'s research group from the University of Oxford has received research grants from the Innovative Medicines Initiative, from Gilead Science, from Theramex and from UCB Biopharma, none of which are related to this manuscript. The remaining authors declare no conflicts of interest.

## Supporting information


**Appendix A1.** The variables that can be calculated using *summariseDrugUtilisation*.

## Data Availability

According to applicable European and national regulations, the data used in this study can only be accessed by the participating researchers after protocol approval. We are therefore not authorised to share or make these data publicly available. The analysis code is openly accessible at: https://github.com/darwin‐eu‐studies/DrugUtilisationArticle. The source code for the DrugUtilisation R package, which contains the dose calculation implementation, is available at: https://github.com/darwin‐eu/DrugUtilisation.
