# Supplementary material for: DrugUtilisation: An R Package to Support Drug Utilisation Research Using the OMOP Common Data Model
Source: Pharmacoepidemiol Drug Saf. 2026 Jul 14;35(7):e70433. doi: 10.1002/pds.70433 (PMC13368618; doi:10.1002/pds.70433)
Supplement: Supplementary file 1 — Appendix A1. The variables that can be calculated using summariseDrugUtilisation. [file PDS-35-e70433-s001.docx]

**Supplementary material**

Appendix A

The variables that can be calculated using *summariseDrugUtilisation* are:

1. ‘numberExposures’: number of drug records included in the analysis. Number of exposures is 3 in Figure 3.
2. ‘numberEras’: number of treatment eras (episodes) included in the analysis, gapEra is used to determine the number of eras. The number of eras is 2 if gapEra is smaller or equal to 4 days, and 1 if gapEra is bigger or equal to 5 days.
3. ‘daysExposed’: number of days that the individual is in a treatment era, as gapEra is used to determine the eras this parameter also affects the days exposed calculation. In Figure 3 the number of days exposed is 42 (26 of the first era and 16 of the second era) if gapEra is smaller or equal to 4 days, otherwise the number of days exposed is 46 days because a single treatment era is considered.
4. ‘daysPrescribed’, sum of the length of each one of the drug records included in the analysis. In figure 3 the number of days prescribed is 48, 21 of the first prescription, 11 of the second one and 16 of the third one).
5. ‘timeToExposure’, number of days till first exposure. In figure 3 the time to exposure is 0 days as the exposure starts on the index date. Time to exposure cannot be negative so even if restrictIncidence is false and exposures that start before the index date are included in the analysis, time to exposure will be 0 in those individuals where exposure starts before index date.
6. ‘initialExposureDuration’, length of the first exposure. In figure 3 the initial exposure duration is 21 days. If there are multiple exposures that start on the first day the longest one is considered.
7. ‘initialQuantity’, quantity associated with the exposures present on the first exposure day. In figure 3 initial quantity is 63. If there are multiple exposures that start in the first day quantities are added up. Note quantity does not have units.
8. ‘cumulativeQuantity’, sum of all the quantity prescribed in the exposures included in the analyses. In figure 3 cumulative quantity is 117. Note quantity does not have units.
9. ‘initialDailyDose’, daily dose at the first day that individual is exposed. In Figure 3 initial daily dose is 1500 mg/day ($\frac{63 \cdot500}{21}$). Note dose can be reported in different units, and then it is reported separately for each one of them.
10. ‘cumulativeDose’, cumulative sum of the doses associated in each one of the exposures. In Figure 3 cumulative dose is 62,200 mg ($63\cdot500+22\cdot300+32\cdot750$). Only dose contribution between index and censor date are considered, e.g. if half of the exposure contribution is outside the index – censor range only half of the cumulative dose is considered for that exposure. Note dose can be reported in different units, and then it is reported separately for each one of them.
